# Assessment of Web-Based Consumer Reviews as a Resource for Drug Performance

**DOI:** 10.2196/jmir.4396

**Published:** 2015-08-28

**Authors:** Swarnaseetha Adusumalli, HueyTyng Lee, Qiangze Hoi, Si-Lin Koo, Iain Beehuat Tan, Pauline Crystal Ng

**Affiliations:** ^1^ Genome Institute of Singapore Singapore Singapore; ^2^ National Cancer Centre Singapore Singapore Singapore; ^3^ Duke-NUS Graduate Medical School Singapore Singapore

**Keywords:** consumer drug reviews, online drug ratings, WebMD, online health websites

## Abstract

**Background:**

Some health websites provide a public forum for consumers to post ratings and reviews on drugs. Drug reviews are easily accessible and comprehensible, unlike clinical trials and published literature. Because the public increasingly uses the Internet as a source of medical information, it is important to know whether such information is reliable.

**Objective:**

We aim to examine whether Web-based consumer drug ratings and reviews can be used as a resource to compare drug performance.

**Methods:**

We analyzed 103,411 consumer-generated reviews on 615 drugs used to treat 249 disease conditions from the health website WebMD. Statistical analysis identified 427 drug pairs from 24 conditions for which two drugs treating the same condition had significantly and substantially different satisfaction ratings (with at least a half-point difference between Web-based ratings and *P*<.01). PubMed and Google Scholar were searched for publications that were assessed for concordance with findings online.

**Results:**

Scientific literature was found for 77 out of the 427 drug pairs and compared to findings online. Nearly two-thirds (48/77, 62%) of the online drug trends with at least a half-point difference in online ratings were supported by published literature (*P*=.02). For a 1-point online rating difference, the concordance rate increased to 68% (15/22) (*P*=.07). The discrepancies between scientific literature and findings online were further examined to obtain more insights into the usability of Web-based consumer-generated reviews. We discovered that (1) drugs with FDA black box warnings or used off-label were rated poorly in Web-based reviews, (2) drugs with addictive properties were rated higher than their counterparts in Web-based reviews, and (3) second-line or alternative drugs were rated higher. In addition, Web-based ratings indicated drug delivery problems. If FDA black box warning labels are used to resolve disagreements between publications and online trends, the concordance rate increases to 71% (55/77) (*P*<.001) for a half-point rating difference and 82% (18/22) for a 1-point rating difference (*P*=.002). Our results suggest that Web-based reviews can be used to inform patients’ drug choices, with certain caveats.

**Conclusions:**

Web-based reviews can be viewed as an orthogonal source of information for consumers, physicians, and drug manufacturers to assess the performance of a drug. However, one should be cautious to rely solely on consumer reviews as ratings can be strongly influenced by the consumer experience.

## Introduction

When choosing among drugs to treat a patient’s condition, clinicians rely on published clinical trials, practice experience, and/or US Food and Drug Administration (FDA) drug labels. However, FDA trial results can be incomplete; 78% of drug trials subject to mandatory reporting did not report their results [1]. Furthermore, published results may be reported in a biased manner, favoring the trial sponsor, which is often also the drug manufacturer [2,3]. Finally, published trials and FDA labels can be challenging to read and inaccessible to patients.

The public is increasingly turning to the Internet for information about drugs and their side effects [4,5]. Three-quarters (73%) of adults with higher education use the Internet for health information [4]. Over a quarter (26%) of Americans read or watch someone else’s experience with health or medical issues online [5], and 16% of Internet users go online to find others who share the same health concerns [5].

Many of the health-related websites, such as WebMD [6] and AskAPatient [7], provide a public forum for consumers to post ratings and reviews on their drug experiences. For example, consumers can submit their reviews of drugs and rate the drugs on a scale of 1-5 at the WebMD website. The websites also ask users to share their disease condition, age, sex, the prescribed duration, and their comments; 3-4% of Internet users have shared their experiences with drugs online [5], which extrapolates to millions of drug experiences.

Various researchers have mined data from health websites to cull useful information from users’ comments [8-14]. Past research on health-related websites and communities has focused on text mining reviewers’ comments. One research group collected consumer reviews of statins from three health-related websites and were able to associate statin consumption with side effects that were not listed by the drug manufacturer but supported by other studies [10]. In another study, consumer reviews and professional drug descriptions reported similar efficacies and adverse effects for two psychotropic drugs [11]. A study on Parkinson’s disease showed that online forums may be a useful source of observational information to complement clinical trials [14]. Past studies have successfully compared drug performance with online resources, but on a case-by-case basis, by focusing on certain classes of drugs or drugs treating a single condition [10-13].

In this study, we investigate if Web-based review ratings can be used as a resource to compare drug performance on a global scale for a comprehensive set of drugs treating a variety of disease conditions. Web-based review ratings potentially provide a fast and easily accessible data source for drugs. We sought to determine if crowd-sourced review ratings are supported by published literature and if they can provide a complementary resource to clinical trials.

##  Methods

### Drug Comparison Based on Web-Based Reviews

Consumer reviews are publicly available and anonymous, so it is ethically acceptable to conduct an analysis of the comments without seeking informed consent from their authors [15]. We obtained an exemption from the *Institutional Review Board* to analyze online consumer-generated reviews.

We downloaded 141,210 reviews of 1503 drugs treating 1123 conditions from WebMD on October 23, 2012. Drug and condition names were taken from the WebMD website. Each review had a user satisfaction rating. The satisfaction rating ranged from 1-5, where 1 is the lowest score for expressing dissatisfaction and 5 is the highest score for expressing satisfaction with the drug. In addition to these ratings, we downloaded the genders and ages of the reviewers and the text comments of the reviews.

We applied pre-processing steps prior to statistical analysis. First, drugs with different modes of deliveries for each individual condition were grouped separately (eg, oral versus intravenous). Second, the reviews of drugs with the same active ingredient(s) were combined. Information about drugs’ brand names and active ingredients was downloaded from the Drugs@FDA database [16]. Of the 1503 drugs on the WebMD drug list, the active ingredients of 920 (61.21%) drugs were identified. For drugs whose active ingredient was not listed in the FDA drug database, the original drug name was kept in the subsequent analysis. Thus, the 1503 drugs were reduced to a total of 1215 groups of active ingredients/drugs, which were used for the analysis. To be concise, we refer to an active ingredient group as a drug in this study. Drugs were required to have at least 30 reviews for the particular condition, and the condition was required to have at least two drug groups to be selected for analysis. This gave a final list of 249 conditions encompassing 615 drugs and 103,411 reviews.

We first tested whether drug ratings were significantly different within a disease condition, before examining drugs individually. We tested at the level of disease condition for two reasons: (1) to control for patient heterogeneity as much as possible, with the assumption that patients taking drugs for the same condition would have similar patient profiles, and (2) because testing for all pairwise drug combinations across all conditions would require a large Bonferroni correction factor, whereas testing for conditions bounds the correction factor to the smaller number of conditions (n=249). Analysis of covariance (ANCOVA) was applied to each condition to determine whether drug(s) account for significant differences in satisfaction ratings while controlling for the covariates of gender and age. For each condition, a linear model was constructed with drug, age, and gender as independent variables, and the satisfaction rating as the dependent variable. Age ranges were transformed into numeric values by taking the mean of the age range (eg, a reviewer with an age range of 25-34 years was assigned 29.5). We computed ANCOVA for the linear model using the “car” library in R (ANOVA, type=“III”). We identified 24 conditions that had a statistically significant difference between their drugs’ ratings (*P*<.05, after Bonferroni correction for the 249 conditions tested).

**Figure 1:**
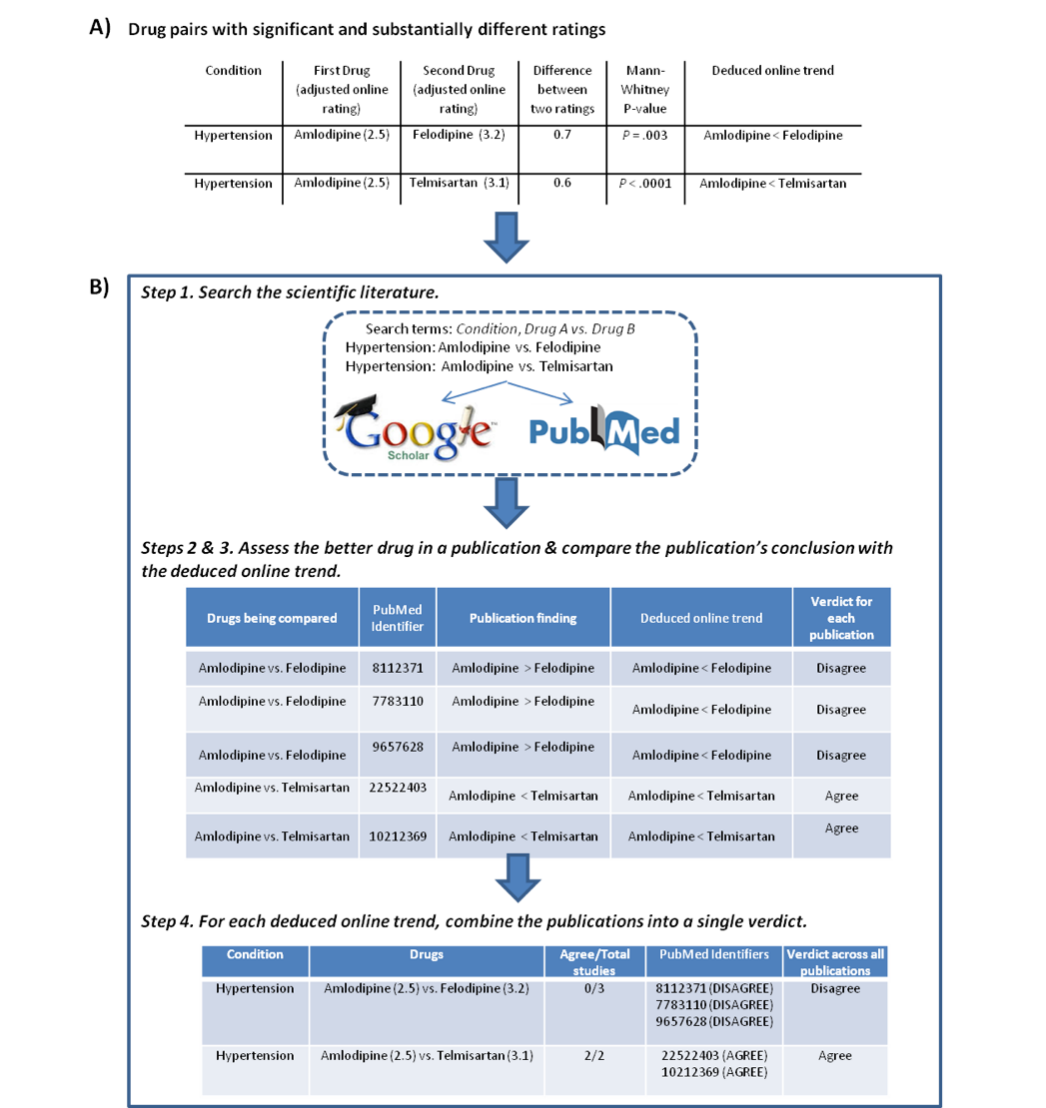
Examples of drug pairs with significant and substantially different ratings, and a procedure flowchart for comparing online findings with scientific literature.

For each of the 24 conditions, we focused on comparing drugs with significant and substantially different ratings. Because comparing two drugs with minor rating differences is difficult, we examined drug pairs where the two drugs’ adjusted drug ratings differed by at least 0.5 points. Adjusted drug ratings are controlled for gender and age because the two drugs may have slightly different patient distributions. An adjusted drug rating is computed by taking the predicted value of the drug’s score for the most common age and gender for the condition (using R’s predict function for the linear model). Additionally, the two drugs were required to have significantly different online satisfaction ratings (Mann-Whitney U test, *P*<.01). In summary, 427 drug pairs with significantly and substantially different ratings were identified from the 24 conditions (see Figure 1 for an example and Multimedia Appendix 1 for the full table). For example, felodipine has an adjusted online rating of 3.2, which is 0.7 points higher than amlodipine (adjusted online rating 2.5), and the two drugs’ online ratings are significantly different from each other (*P*=.003). Online trends can be deduced from each drug pair. In the aforementioned example, the deduced online trend is that felodipine is a better drug than amlodipine. Thus, 427 online trends are deduced from the 427 drug pairs.

### Comparison With Literature

#### Overview

The aim of this study is to see whether scientific literature supports the deduced online trends. This was done by mining the literature for a comparison of the two drugs from that particular online trend. Figure 1 shows an overview of the steps, using three drugs that treat hypertension as an example.

#### Step 1. Search the Scientific Literature

Literature searches were carried out for all 427 drug pairs with significantly and substantially different Web-based ratings. Because the WebMD condition’s name may not be standard, MedDRA’s preferred term for the condition name was used for the literature search [17] (see Multimedia Appendix 2 for condition mappings). The literature searches were conducted on Google Scholar and PubMed. Each search was limited to three publications for practicality, so the maximum number of publications each pairwise drug comparison could have is six (three for Google Scholar and three for PubMed). Occasionally, official regulatory bodies such as the FDA were also used as a source because their documents would be discovered by Google Scholar (eg, see Armour Thyroid vs levothyroxine; Multimedia Appendix 3).

Publications were required to have the drug name exactly and treat the same condition as the WebMD listing. We discarded publications that did not pertain to humans (eg, studies in rats) and case reports on single patients. For 82 out of the 427 pairwise comparisons, we found 152 pieces of scientific literature (132 head-to-head comparisons, 11 reviews, three meta-analyses, and six others).

#### Step 2. Assess the Better Drug in the Publication

The better performing drug was interpreted from a publication’s abstract. Two authors read the abstract and decided whether one drug performed better than the other, if the two drugs performed similarly, or if performance was unclear. An example of an unclear performance is if drug A is more effective but has worse side effects than drug B. If two authors disagreed on the classification, the abstract was discussed between the 2 authors until an agreement was reached. The decision-making process for head-to-head comparisons, meta-analyses, regulatory bodies, and review articles was identical.

#### Step 3. Compare a Publication’s Conclusion With the Corresponding Online Trend

A verdict was determined as to whether the better drug from a publication concurred with the better drug from the corresponding online trend. We classified each publication as “agree” when the publication’s abstract agreed with the online trend and “disagree” when the publication disagreed with the online trend (see Figure 1). An example of an “agree” publication is a paper that states: “Telmisartan was more effective than amlodipine in preventing AF [atrial fibrillation] recurrences” [18]. According to WebMD, telmisartan has a higher Web-based rating than amlodipine (3.1 vs 2.5). Therefore, the publication agrees with the online trend that telmisartan is the better drug. A “disagree” verdict is given when the publication states that the poorly rated WebMD drug has better or similar performance, or if the publication’s conclusion was unclear.

#### Step 4. For Each Deduced Online Trend, Combine the Publications Into a Single Verdict

Because a single online trend can have multiple publications, the publications’ agree/disagree statuses are summarized into a single verdict. The verdict was concluded as “agree” when the majority of the publications for that comparison agreed with the deduced online trend. The verdict was concluded to “disagree” when the majority of publications disagreed with the deduced online trend. For example, if a drug comparison had four published studies, of which three agreed with the deduced online trend and one did not, we concluded the verdict to “agree” with the deduced online trend. For five pairwise drug comparisons, an equal number of publications agreed and disagreed with WebMD ratings; these inconclusive drug comparisons were removed from consideration. In total, there were 77 online trends with .50 point difference that had verdicts summarized from 141 pieces of scientific literature. For these 77 online trends, 48 and 29 had “agree” and “disagree” verdicts with the scientific literature, respectively (Multimedia Appendix 3). Most (71/77, 92%) of the verdicts were unanimous verdicts, where all the publications agreed with each other as to which was the better drug (Multimedia Appendix 3).

To determine if the observed number of “agree” verdicts was more than expected by chance, the *P* value for publication support was calculated by assuming a 0.50 probability of “agree” verdicts and a 0.50 probability of “disagree” verdicts. The probability of observing at least *n* number of “agree” verdicts was calculated using a cumulative binomial distribution.

### Information Extraction From Food and Drug Administration Labels

FDA labels were used to reconcile the disagree verdicts between publications and deduced online trends. A drug’s FDA label was used to determine the drug’s serious side effects, off-label use, and addictive properties. To investigate serious side effects, we inspected a drug’s FDA label for a black box warning, which is the strictest warning by the FDA. To see whether a drug is being used off-label, we looked at the conditions listed under the “Indications and usage” section. If the WebMD/MedDRA condition was not listed in the Indications section, we deemed this “off-label” use. To identify drugs with addictive properties, we inspected if a drug’s FDA label noted drug abuse and dependence as a side effect.

The purpose of examining FDA labels was to find differences between drugs. If both of the drugs in the pairwise comparison had black box warnings or both drugs had addictive properties, this was not recorded as an observation because the two drugs were similar for that aspect.

### Text Mining of Reviews

For some drugs, we examined the reviewers’ comments to hypothesize why publications and deduced online trends might disagree. Frequencies of certain words were counted in the comment section of reviews. The number of drug reviews that contained the term was divided by the total number of drug reviews. Statistical significance for difference in word frequencies was calculated using the chi-square test.

For type 2 diabetes, reviewers’ comments were searched for the word “heart” because the poorly rated drug pioglitazone had a black box warning for congestive heart failure. When looking at addictive drugs (carisoprodol, nefazodone hydrochloride, and diazepam), reviewers’ comments were searched for the words “abuse” and “addict”.

For asthma, we found the most frequent words among reviewers’ comments by using word clouds. Reviewers’ comments were fed to Voyant Tools [19], which removes stop words from the reviews and generates a word cloud from the remaining words. For the drug ProAir, “inhaler” was the most frequent word. For albuterol, ProAir’s generic equivalent, “asthma” was the most frequent word.

## Results

### Drug Differences Identified From Web-Based Reviews

Our study investigates the usefulness of Web-based rating differences between drugs. Over 140,000 drug reviews were downloaded from WebMD. To detect drug rating differences, ANCOVA analysis was applied to 249 disease conditions, of which 24 had different performances between drugs (see Methods). Within the 24 conditions, there were 427 drug pairs that had substantially and significantly different ratings, with at least .50 point difference between the two drugs’ ratings (*P*<.01 Mann-Whitney) (see Multimedia Appendix 1 and Methods).

For each drug pair, one can deduce an online trend because one drug rates significantly higher than the other drug. For example, felodipine has a higher online rating than amlodipine (3.2 vs 2.5, *P*=.003), so the deduced online trend is that felodipine is a better drug than amlodipine. Examples for two pairwise drug comparisons pertaining to the condition hypertension are found in Figure 1.

**Figure 2:**
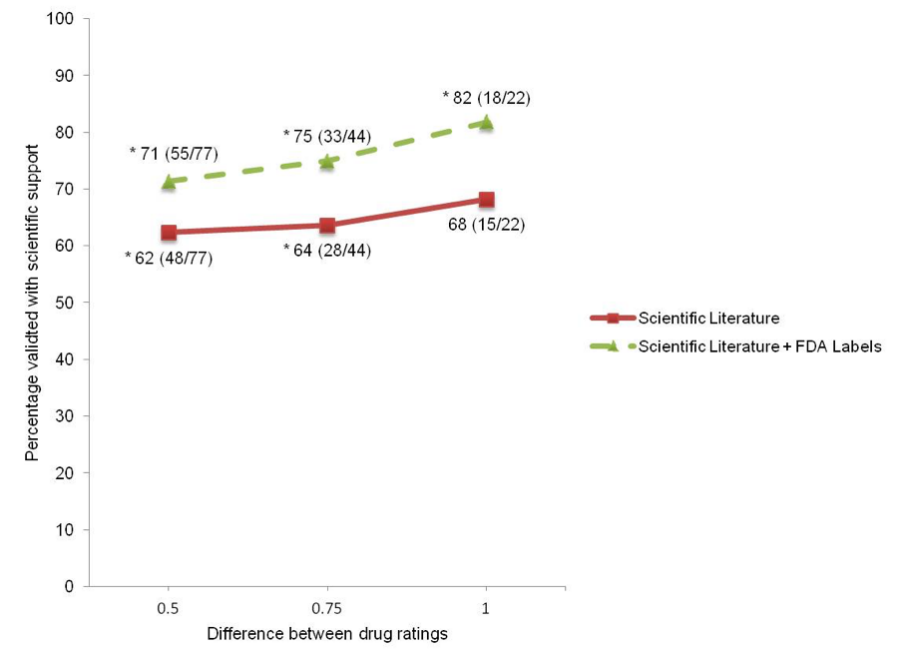
Concordance between deduced online trends and scientific support at varying levels of point differences between 2 drugs’ online ratings. The solid line indicates the concordance of online trends with literature and the dashed line indicates the concordance of online trends with scientific literature and FDA labels. For each data point, the percentage concordance is shown and the number of drug pairwise comparisons agreeing with scientific support divided by the total number of drug pairwise comparisons are given in parenthesis. The asterisk indicates statistical significance with P<.05 according to the binomial test.

To assess if deduced online trends were concordant with scientific literature, we manually searched PubMed and Google Scholar for publications that compare the two drugs belonging to the online trend (see Methods and Figure 1). A verdict was determined as to whether the majority of the publications agreed with the online drug trend. Verdicts were determined for 77 (18.0%) of the 427 drug pairs (see examples in Figure 1 and the full table in Multimedia Appendix 3). Summarizing across the 77 pairwise drug comparisons with at least a half-point rating difference, 62% (48/77) of the literature verdicts are concordant with their deduced online trends (*P*=.02, binomial distribution) (Figure 2). When raising the cutoff to a 1-point rating difference between two drugs, the concordance rate between Web-based ratings and literature increases to 68% (15/22). The result for the higher cutoff is not significant, most likely due to low numbers (*P*=.07) (Figure 2).

### Investigating Online Trends That Are Discordant With Scientific Literature

While the majority of deduced online trends were in concordance with the literature, 38% (29/77) were not. We investigated why scientific literature was not consistent with Web-based ratings. We observed that (1) drugs with FDA boxed warnings or used off-label for the WebMD condition rated poorly among online reviews, (2) drugs with addictive properties had higher review ratings, and (3) patients rated alternative treatments higher. A problem with drug delivery was also discovered independently. The summary of these findings can be found in Table 1 (with additional details in Multimedia Appendix 4) and is further elaborated on in the following text.

**Table 1:** Summary of observations for drug comparisons where Web-based ratings disagreed with publications.

		Number of drug comparisons
**Consistent with FDA label**		
	Drug with boxed warning rated lower	7
	Drug used off-label rated lower	2
Addictive drug rated higher		5
Alternative or second-line drug rated higher		2
Unexplained		13
Total		29

### Poorly Rated Drugs Have Food and Drug Administration Black Box Warnings

FDA drug labels can have black box warnings that inform of serious side effects. For seven drug comparisons, the drugs with FDA black box warnings were poorly rated among Web-based reviews even though they performed better according to publications (Multimedia Appendix 4). The corresponding competing drugs in the pairwise comparison did not have black box warnings. For example, the type 2 diabetes drug pioglitazone hydrochloride has a black box warning of increased risk of congestive heart failure [20]. Approximately 9.9% (56/568) of pioglitazone hydrochloride reviewers complained of heart problems, compared with 3.08% (55/1788) of the complaints from other drugs used to treat type 2 diabetes (chi-square *P*<.001) (see Methods). Sample comments for pioglitazone hydrochloride are “it is a killer!!! better choices are available. who needs bladder cancer or heart problems!!!” and “had very bad chest pains thought i was having heart failure. chest pains stop[p]ed within 3 days of stop[p]ing the drug will not try it again. it did lower blood sugars”. Thus poor Web-based ratings for certain drugs are supported by FDA black box warnings.

If one assumes FDA black box warnings are accurate and authoritative compared to scientific publications (which can be biased [2,3]), then the seven discordant comparisons may be considered correct for the deduced online trends. If we re-classify these seven to a verdict of “agreed”, then the support rate of online ratings is 71% ((48+7)/77) (*P*<.001) (see dashed line in Figure 2). This is an overestimate of the support rate because we investigated only pairwise comparisons with “disagree” verdicts. Future studies should include a more thorough analysis examining FDA labels for all drugs.

### Off-Label Drug Use Is Rated Lower

Drugs are sometimes used to treat a condition that has not been approved by the FDA. The practice of off-label drug use is prevalent [21]. For two comparisons, the drug with a lower Web-based rating was not FDA-approved for the WebMD condition (Multimedia Appendix 4). For example, alprazolam has the highest rating compared to all other drugs for panic disorder and is one of the few drugs indicated for panic disorder on its FDA label. Most of the other drugs that have reviews on WebMD for panic disorder are indicated to treat depression (eg, citalopram) or anxiety disorder (eg, diazepam), and these other drugs have lower Web-based ratings. Therefore, even though the practice of off-label drug prescription is common, Web-based reviews can reveal user dissatisfaction with off-label drugs.

### Addictive Drugs Are Highly Rated

Three drugs (diazepam for treating anxiety and muscle spasms, nefazodone hydrochloride for treating depression, and carisoprodol for treating muscle spasms) are addictive according to FDA labels. These drugs have poor performances according to the scientific literature, but higher Web-based ratings compared to other drugs treating the same condition. This suggests the possibility that patients may rate drugs with addictive properties higher.

For example, carisoprodol (adjusted rating 4.23) is rated higher than the other drugs that treat muscle spasm (Multimedia Appendix 1). Head-to-head comparisons showed that carisoprodol and cyclobenzaprine (adjusted rating 3.26) perform similarly, but carisoprodol’s usefulness is mitigated by its potential for abuse [22]. The drug has an FDA label that warns of its addictive properties. Interestingly, 12.6% (28/223) of the reviewers’ comments for carisoprodol contained the word “addict” or “abuse”, compared with 0% (0/371) for cyclobenzaprine reviews (*P*<.001) (see Methods). However, even though carisoprodol reviewers recognized the potential for abuse, they rated carisoprodol highly: 68% (19/28) of the reviewers that mentioned addictiveness gave a satisfaction score of 4 or higher. Some sample comments are “It’s a great medication but can easily become dangerously addicting” and “so far this has been the best medication to help give me almost complete relief. Just be careful using it, it is addictive.”

Similarly, for the addictive drug diazepam, 87% (13/15) of the reviewers for anxiety and muscle spasm that mentioned “addict” or “abuse” still gave ratings 4 or higher. This suggests that patients, despite being aware of a drug’s potential for abuse, will still rate an addictive drug high. It highlights the importance of professional medical advice and FDA labels, and a caution when relying on consumer-generated reviews. Another possible explanation for why drugs with addictive properties are rated higher may be due to stronger drug efficacy and potency or psychoactive properties. A more systematic study of the impact on addictiveness in Web-based ratings should be conducted to see if these observations can be generalized.

### Alternative Treatments Can Have Higher Ratings

Drug accessibility and past experience may influence reviewers’ drug ratings. There were two drug comparisons for which an alternative or second-line drug was rated higher than the commonly prescribed first-line drug (Multimedia Appendix 4). For hypothyroidism, online patients expressed greater satisfaction with the drug Armour (adjusted rating 3.92) than levothyroxine (adjusted rating 2.22) (Multimedia Appendix 1). Treatment with Armour is highly controversial. Armour is desiccated animal thyroid, and this natural treatment has been used to treat hypothyroidism since the 1890s [23], prior to the formation of the FDA. Levothyroxine is a synthetic form of the thyroid hormone and is FDA-approved [24-26]. Despite professional endorsements of the synthetic form because of its better stability and quality assurance (by United States FDA, Endocrine Society of Australia, and British Thyroid Association), there is a grassroots movement in support of using naturally desiccated thyroid [27]. Head-to-head comparisons exist, but interpretations of these comparisons are controversial [28]. In surveys, patients preferred natural desiccated thyroid over thyroxine alone [28], and the majority of patients who had tried conventional therapies but then switched to natural desiccated thyroid were more satisfied with the natural treatment [29]. Web-based patient reviews are consistent with surveys rather than the professional recommendations, and the higher rating may be due to subgroups that are satisfied with Armour as an alternative treatment.

These results suggest ratings can be influenced by a reviewer’s treatment history. If the first line of treatment is ineffective and the alternative treatment provides relief but is harder to obtain, reviewers may compensate with higher ratings for the alternative/second-line drug to confirm that the less popular or less common choice was effective for them. A more systematic study is necessary to see if this trend can be generalized.

In summary, Web-based ratings that disagree with scientific literature can be explained by (1) drugs with FDA boxed warnings rating poorly, (2) drugs used for off-label conditions rating poorly, (3) drugs with addictive properties rating higher, and (4) alternative treatments rating higher. These explanations account for over half (16/29) of the discordances between literature and deduced online trends (Table 1). The remaining 13 disagreements were designated as “Unexplained”. Further investigation is needed to reconcile the remaining cases.

### Drug Delivery Design

Web-based reviews can lead to new findings; a drug delivery issue for an asthma inhaler was discovered. This came to our attention because the asthma inhaler ProAir had low Web-based ratings (average rating 1.46), yet its generic equivalent albuterol had high Web-based ratings (average rating 3.48). We observed this strange phenomenon when we had not yet combined the brand-name ProAir with its generic equivalent albuterol. To understand this unexpected discrepancy, we inspected the text of the reviews. The most frequent word in the ProAir reviews is “inhaler”, suggesting that dissatisfaction with ProAir was due to the inhaler’s design. Some comments on the inhaler include: “This inhaler continually clogs and I waste quite a bit of medication” and “ProAir frequently clogs and never really seems to dispense properly. Its effectiveness is a large step backwards from fast acting inhalers 10 years ago”.

The company responded by releasing a newly designed inhaler in 2012, which included a dosage counter capable of tracking the number of doses remaining in the inhaler [30]. Findings related to drug-delivery issues may not be assessed in clinical trials. Therefore, consumer input from Web-based reviews can extend beyond the efficacy of the active ingredient and can benefit the drug manufacturer.

## Discussion

### Principal Findings

Previous publications have studied drug reviews using online resources, but these approaches tend to examine drugs on a case-by-case basis [10-13]. To the best of our knowledge, this is the first study to analyze online drug satisfaction on a global scale for a comprehensive set of drugs across many disease conditions. We found 427 significantly different drug pairs where the drugs’ ratings had more than a half-point difference. For 77 of the drug pairs, we determined whether the scientific literature agreed or disagreed with the deduced online trends. For a 0.5-point rating difference, 62% (48/77) of the deduced online trends were concordant with scientific literature (*P*=.02). The concordance increased to 68% (15/22) when drug pairs with a larger rating difference (at least 1-point) were considered, but this was not statistically significant (*P*=.07), possibly due to small sample size. Further investigation of the remaining 29 that were discordant showed that seven inferred online findings were supported by FDA labels. Lower-rated drugs had FDA black box warnings indicating serious side effects. If one were to include the FDA black box warnings as supportive evidence for the deduced online trends, the scientific support for online trends increases to 71% (55/77) (*P*<.001).

Examination of the discordant drug comparisons suggested reviewers may be rating addictive drugs and alternative drugs higher. Addictive and alternative drugs may have similar efficacy to non-addictive and standard drugs; high ratings could be an artifact of users’ subjectivity. These observations were found for a small number of drug comparisons and may be anecdotal. A more comprehensive study is necessary before generalizing if addictive drugs or second-line drugs tend to have higher ratings.

Web-based reviews also uncovered a new finding: the suboptimal design of an asthma inhaler. Such analyses can assess the satisfaction of a drug beyond the efficacy of its active ingredient as features like drug delivery may not always be assessed in clinical trials. A drug manufacturer can use this knowledge to improve the delivery design and manufacturing process.

### Limitations

The use of Web-based reviews is independent, fast, and inexpensive, but it also poses some challenges. The reviewers themselves may be biased. People who write reviews may be different from the general population. Reviewers provide a subjective rating on “satisfaction” and do not have objective criteria to assess clinical benefit, unlike the “harder” endpoints that are evaluated in clinical studies. Users experience a drug’s effects on a broader spectrum than the narrowly defined efficacy endpoints of clinical drug studies. This could cause the differences between quantitative Web-based ratings and published drug efficacies. Our study also suggests that reviewers may downplay certain side effects, such as addictiveness. Another disadvantage is that most review websites do not require information on important clinical input variables such as dosages, drug compliance, duration of treatment, additional drugs taken, strict diagnostic criteria, uniform disease severity/stage, smoking status, and general health. Therefore, one cannot ensure that the patients receiving drug A have similar medical profiles to those receiving drug B. While an analysis based on consumer reviews may involve a certain degree of bias and caveats, it also measures the exposure of a drug in a more realistic and diverse setting. Another limitation of our study is that we used only one source for reviews; future work will be incorporation of other additional online sources.

### Conclusion

A small number (3-4%) of Internet users have shared their experiences with drugs online [5], which extrapolates to millions of drug experiences. The large size and broad accessibility of this database has an advantage over controlled clinical trials that recruit a finite number of patients. This is counterbalanced by the fact that Internet users represent a diverse population, in contrast to controlled clinical trials, which consist of homogeneous patients who meet strict trial inclusion criteria. Nevertheless, our study characterizes the use of Web-based reviews for comparing performances of drugs. In conclusion, we have shown that consumer reviews can be used as an orthogonal source to reveal insights on drug performance.

## Appendix

Multimedia Appendix 1 Literature searches for 427 drug pairs where the two drugs have substantially different online ratings that are significantly different. The adjusted online drug ratings are shown in columns 4 and 5, and P values for Mann-Whitney test are in column 6.

Multimedia Appendix 2 Search terms for the 24 conditions with drug differences.

Multimedia Appendix 3 Drug pairs for which scientific literature was found, and their verdicts as to whether the scientific literature agreed or disagreed with online trends. The second column shows the two drugs being compared with their adjusted online ratings in parentheses. The third column shows the scientific literature that compares the two drugs, and whether it disagrees or agrees with the deduced online finding in parentheses. The fourth column lists the number of scientific studies that agree with the deduced online finding out of the total number of relevant scientific studies. An overall verdict summarizing the multiple scientific studies is shown in the fifth column. The sixth column specifies whether the verdict is unanimous. The last column lists the publication type for each piece of scientific literature.

Multimedia Appendix 4 Possible explanations for cases where scientific literature disagrees with deduced online findings.

## Abbreviations

ANCOVA: analysis of covariance
ANOVA: analysis of variance
FDA: Food and Drug Administration
