# Supplementary material for: Assessment of Web-Based Consumer Reviews as a Resource for Drug Performance
Source: J Med Internet Res. 2015 Aug 28;17(8):e211. doi: 10.2196/jmir.4396 (PMC4642403; doi:10.2196/jmir.4396)
Supplement: Multimedia Appendix 2 [file jmir_v17i8e211_app2.pdf]

Multimedia Appendix 2. Search terms for the 24 conditions with drug differences. The first column lists the condition name according to WebMD. Because WebMD condition's name may not be standard, MedDRA's "preferred term" for the condition was used (ww.meddra.org).

**WebMD Condition Name**	**MedDRA Condition Name**
Anxious	Anxiety
Asthma:Inhaler	Asthma
Atrial Fibrillation	Atrial fibrillation
Attention Deficit Disorder with Hyperactivity	Attention deficit/hyperactivity disorder
Birth Control	Birth control
Change of Life Signs	Change of life signs / quality of life
Chronic Pain	Chronic pain
Chronic Trouble Sleeping	Insomnia
Condition in which Stomach Acid is Pushed Into the Esophagus	Gastro oesophageal reflux disease
Depression	Depression
High Blood Pressure	Hypertension
Joint Damage causing Pain and Loss of Function	Arthritis
Major Depressive Disorder	Major depression
Migraine headache	Migraine headache
Muscle Spasm	Muscle spasms
Nausea and Vomiting	Nausea and vomiting
Overweight	Obesity
Pain	Pain/acute pain
Panic Disorder	Panic disorder
Repeated Episodes of Anxiety	Repeated episodes of anxiety
Rheumatoid Arthritis	Rheumatoid arthritis
Stop Smoking	Smoking cessation
Type 2 Diabetes Mellitus	Type 2 diabetes mellitus
Underactive Thyroid	Hypothyroidism
