# Supplementary material for: Assessment of Web-Based Consumer Reviews as a Resource for Drug Performance
Source: J Med Internet Res. 2015 Aug 28;17(8):e211. doi: 10.2196/jmir.4396 (PMC4642403; doi:10.2196/jmir.4396)
Supplement: Multimedia Appendix 4 [file jmir_v17i8e211_app4.pdf]

Multimedia Appendix 4. Possible explanations for cases where scientific literature disagrees with deduced online findings. The possible explanation is shown in the first column and the drug with the corresponding property in the second column. The deduced online trend which contains the drug is in the third column, and this trend is not supported by scientific literature (see Multimedia Appendix 3).

**Possible explanation**	**Drug**	**Deduced Online Trend**	**Medical condition**
Drug with boxed warning rated lower			
	Aliskiren hemifumarate	Aliskiren hemifumarate (2.41) < Atenolol (3.28)	Hypertension
	Losartan	Losartan (2.41) < Felodipine (3.2)	Hypertension
	Metoclopramide hydrochloride	Metoclopramide hydrochloride (2.53) < Promethazine hydrochloride (4.01)	Nausea and vomiting
	Morphine sulfate; naltrexone hydrochloride	Morphine sulfate; naltrexone hydrochloride (2.34) < Morphine sulfate (3.7)	Chronic pain
	Pioglitazone hydrochloride	Pioglitazone hydrochloride (2.38) < Glimepiride (3.03)	Type 2 diabetes mellitus
	Pioglitazone hydrochloride	Pioglitazone hydrochloride (2.38) < Glyburide (2.99)	Type 2 diabetes mellitus
	Prochlorperazine maleate	Prochlorperazine maleate (2.62) < Promethazine hydrochloride (4.01)	Nausea and vomiting
Drug used off-label rated lower			
	Bupropion hydrochloride	Bupropion hydrochloride (2.83) < Methylphenidate hydrochloride (3.44)	Attention deficit/hyperactivity disorder
	Citalopram hydrobromide	Alprazolam (4.23) > Citalopram hydrobromide (3.52)	Panic disorder
Addictive drug rated higher			
	Carisoprodol	Carisoprodol (4.23) > Metaxalone (3.13)	Muscle Spasms
	Diazepam	Hydroxyzine hydrochloride (2.71) < Diazepam (4.26)	Anxiety
	Diazepam	Diazepam (3.97) > Tizanidine (2.97)	Muscle Spasms
	Diazepam	Diazepam (3.97) > Tizanidine hydrochloride (3.24)	Muscle Spasms
	Nefazodone hydrochloride	Nefazodone hydrochloride	Depression

		(3.88) > amitriptyline (3.06)	
Alternative treatment rated higher			
	Armour thyroid	Armour thyroid (3.92) > Levothyroxine (2.72)	Hypothyroidism
Second-line drug rated higher			
	Triazolam	Zaleplon (2.25) < Triazolam (3.88)	Insomnia
